# Pathways and Networks-Based Analysis of Candidate Genes Associated with Nicotine Addiction

**DOI:** 10.1371/journal.pone.0127438

**Published:** 2015-05-12

**Authors:** Meng Liu, Rui Fan, Xinhua Liu, Feng Cheng, Ju Wang

**Affiliations:** 1 School of Biomedical Engineering, Tianjin Medical University, Tianjin, China; 2 Department of Pharmaceutical Science, College of Pharmacy, University of South Florida, Tampa, Florida, United States of America; Yale University, UNITED STATES

## Abstract

Nicotine is the addictive substance in tobacco and it has a broad impact on both the central and peripheral nervous systems. Over the past decades, an increasing number of genes potentially involved in nicotine addiction have been identified by different technical approaches. However, the molecular mechanisms underlying nicotine addiction remain largely unclear. Under such situation, a comprehensive analysis focusing on the overall functional characteristics of these genes, as well as how they interact with each other will provide us valuable information to understand nicotine addiction. In this study, we presented a systematic analysis on nicotine addiction-related genes to identify the major underlying biological themes. Functional analysis revealed that biological processes and biochemical pathways related to neurodevelopment, immune system and metabolism were significantly enriched in the nicotine addiction-related genes. By extracting the nicotine addiction-specific subnetwork, a number of novel genes associated with addiction were identified. Moreover, we constructed a schematic molecular network for nicotine addiction via integrating the pathways and network, providing an intuitional view to understand the development of nicotine addiction. Pathway and network analysis indicated that the biological processes related to nicotine addiction were complex. Results from our work may have important implications for understanding the molecular mechanism underlying nicotine addiction.

## Introduction

Cigarette smoking is a worldwide epidemic, and one of the major preventable causes of morbidity and mortality [1–2]. Although there are some effective control policies and interventions, the negative effect of tobacco abuse on public health and social economy is still astonishing, highlighting the need for continuing efforts. According to World Health Organization (WHO), currently there are about 1.3 billion smokers worldwide, most of whom come from the low- or middle-income countries; and it is estimated that more than 5 million smokers die from smoking-related diseases every year [3–4]. If effective measures are not adopted, by 2020, smoking will become the biggest health problem worldwide, and the number of deaths caused by smoking will reach 10 million per year [5]. Besides the health problems, smoking also causes heavy economic burden on society. According to the Centers for Disease Control and Prevention (CDC), in USA alone, the economic burden made by smoking to society, including both the direct health care expenditures and the loss of productivity, can be as high as $193 billion a year [6]. Therefore, developing effective approaches and drugs for the treatment and prevention of smoking are of huge challenge in public health.

Nicotine, as the primary psychoactive component of tobacco smoke, binds to neuronal nicotinic acetylcholine receptors (nAChRs), a family of ligand-gated ion channels [7], facilitating various neurotransmitter release such as dopamine, glutamate, serotonin and γ-aminobutyric acid (GABA) [8–10] and thereby producing a number of neurophysiological and behavioral effects. Emerging evidence suggests that repeated exposure to nicotine can alter the level or types of genes expressed in multiple brain regions and such alteration ultimately mediates the functions of the related neurons and neural circuits. Numerous studies aiming to discover genetic variants or candidate genes, such as genome-wide association studies, genome-wide linkage scan, gene expression and candidate gene association studies, have found a large number of promising genes and chromosomal regions involved in the etiology of nicotine addiction [11–13]. Moreover, various neural pathways and transmitter systems have emerged as compelling candidates for the processing of addictive properties of nicotine, which provide a valuable resource to unravel the molecular mechanism underlying nicotine addiction. Through its interaction directly or indirectly with these genes and biological pathways, nicotine evokes multiple effects in the central nervous system.

During the past decade, rapid advances in high-throughput technologies have brought unprecedented opportunities for the large-scale analysis of the nicotine addiction-related genes/proteins, leading to a rapid generation of large-scale nicotine addiction-related data. These datasets are often heterogeneous and multi-dimensional, which makes integrating and arranging such datasets to ascertain the key molecular mechanisms and to transform the data into meaningful biological phenomenon a major task and challenge. To meet the demand, pathway and network-based analyses have become an important and powerful approach to elucidate the biological implications underlying complex diseases [14–16]. Such a systems biology approach could be pivotal for better understanding of mental disorder at the molecular level [17]. Thus, a comprehensive analysis of the nicotine addiction-related candidate genes within a systematic framework may provide us important insights on the molecular mechanisms underlying nicotine addiction.

In this study, we performed a systematic analysis on genes potentially involved in nicotine addiction by identifying the enriched functional categories and pathways, as well as examining the crosstalk among the significantly enriched pathways. Then, we extracted a nicotine addiction-specific network and constructed a molecular network of nicotine addiction.

## Materials and Methods

### Data sources

In this study, the candidate genes for nicotine addiction included 220 genes prioritized via a multi-source-based gene approach [18]. Briefly, genes identified to be related to nicotine addiction or involved in the physiological response to nicotine exposure or smoking behaviors were collected from different sources, including genetic association analysis, genetic linkage analysis, high throughput gene/protein expression analysis and/or literature search of single gene/protein-based studies. Based on these resources, the 11,781 genes collected were scored and a weight value was determined for each category. The overall relation between a gene and nicotine addiction was measured by a combined score derived from its scores in the four categories. Then, the genes were ranked according to the combined scores with a larger score value indicating a potentially higher correlation between the gene and nicotine addiction. Based on the distribution of the combined score of all the genes, 220 genes on the top of the list (i.e., with the largest combined scores) were selected as the prioritized nicotine addiction-related genes (NAGenes).

The human protein-protein interaction (PPI) data were downloaded from the Protein Interaction Network Analysis (PINA) platform (May 21, 2014) [19], which integrated data from six major public PPI databases, namely IntAct, BioGRID, MINT, DIP, HPRD, and MIPS/MPact. Also, we downloaded the related annotation files from NCBI (ftp://ftp.ncbi.nlm.nih.gov/gene/) (May 24, 2014), including the Entrez gene information database of human (Homo_sapiens.gene_info.gz), the dataset specifying relationship between pairs of NCBI and UniProtKB protein accessions (gene_refseq_uniprotkb_collab.dz), and file containing mappings of Entrez Gene records to Entrez RefSeq Nucleotide sequence records (gene2refseq.gz). Then the human PPI data were mapped to NCBI human protein-coding genes and the unmapped proteins were discarded. After removing self-interactions and redundant interacting pairs, a final human PPI network containing 15,093 nodes and 161,419 edges was obtained.

### Functional enrichment analysis

To examine the functional features of NAGenes, WebGestalt [20] and Ingenuity Pathway Analysis system (IPA; https://analysis.ingenuity.com) were applied for functional enrichment analysis, including Gene Ontology (GO) term analysis and pathway analysis. WebGestalt is a web-based integrated data mining system to evaluate the significance of GO terms enrichment in the candidate genes. IPA is designed to identify global canonical pathways from a given list of genes. Basically, the genes with their symbol and/or corresponding GenBank Accession Numbers are uploaded into the IPA and compared with the genes included in each canonical pathway. All the pathways with one or more genes overlapping the candidate genes are extracted, with each of them assigned a *p* value to denote the probability of overlap between the pathway and the input genes via Fisher’s exact test. Then, the corresponding multiple testing correction p-value is calculated with the method of Benjamini and Hockberg, namely P_BH_-value [21].

### Pathway crosstalk

We further performed pathway crosstalk analysis to explore the interactions among significantly enriched pathways. To describe the overlap between any given pair of pathways, we introduced two measurements, i.e., the Jaccard Coefficient $(\text{JC})=\left|\frac{A\cap B}{A\cup B}\right|$ and the Overlap Coefficient $(\text{OC})=\frac{\left|A\cap B\right|}{\text{min}\left(\left|A\right|,\left|B\right|\right)}$, where A and B are the lists of genes included in the two tested pathways. To construct the pathway crosstalk, we implemented the following procedure:

1. Select a set of pathways for crosstalk analysis. Only the pathways with P_BH_-value less than 0.01 were used. Meanwhile, the pathways containing less than 5 candidate genes were removed because pathways with too few genes may have insufficient biological information.
2. Count the number of shared candidate genes between any pair of pathways. Pathway pair with less than 3 overlapped genes was removed.
3. Calculate the overlap of all pathway pairs and rank them. All the pathway pairs were ranked according to their JC and OC values.
4. Visualize the selected pathway crosstalk. To focus on the most important biological themes, we only chose those crosstalks with scores in the top 20% using the software Cytoscape [22]. Of note, this criterion was set up somewhat arbitrarily, but the results were well displayed with an appropriate number of nodes (pathways) and edges (crosstalk). We also used other criteria to select the crosstalks among pathways, but the results were similar.

### Construction of nicotine addiction-specific network

Steiner minimal tree algorithm uses a greedy search strategy to merge the smaller trees into larger ones until only one tree connecting all input seeds is built [23]. We applied GenRev [24], a network-based software package to search the optimal intermediate nodes (genes) for the connection of input seed genes via the Steiner minimal tree algorithm, to extract a subnetwork from the human PPI network by using the 220 NAGenes as seeds. To test the non-randomness of this subnetwork, we first generated 1000 random networks with the same number of nodes and edges as the nicotine addiction-specific network using Erdos-Renyi model in R igraph package [25]. Then, we calculated the average values of the shortest-path distance and clustering coefficient. By counting the number of random networks with average shortest-path distance (n_L_) smaller than that of the nicotine addiction-specific network and the number of random networks with average clustering coefficient (n_C_) higher than the observed clustering coefficient, we were able to estimate the significance of non-randomness. Finally, we calculated the empirical p-value = n_L_/1000 and n_C_/1000, respectively.

## Results

### GO enrichment analysis in nicotine addiction-related genes

The nicotine addiction-related genes (NAGenes) were involved in diverse biological functions (S1 Table). For example, some genes were related to synaptic transmission, such as the nicotinic cholinergic receptors (e.g., CHRNA1, CHRNA4, CHRNA7, CHRNA10, and CHRNB2) and dopamine receptors (DRD1, DRD2, DRD3, DRD4 and DRD5); some genes were involved in drug metabolism, such as sulfotransferase 1A1 (SULT1A1), alcohol dehydrogenase 1B (ADH1B), aldehyde dehydrogenase 2 (ALDH2), cytochrome P450 17A1 (CYP17A1) and CYP1A1; some genes were related to cellular transport, e.g., solute carrier family 18 (vesicular monoamine) member 2 (SLC18A2), solute carrier family 6 (neurotransmitter transporter, serotonin) member 4 (SLC6A4) and solute carrier family 9 (sodium/hydrogen exchanger) member 9 (SLC9A9).

Functional enrichment analysis revealed a more specific function pattern of these genes. Among the GO terms significantly enriched in the candidate genes (Table 1), including those associated with neurodevelopment or synaptic transmission. In the biological process, terms directly related to neurodevelopment, e.g., synaptic transmission (P_BH_ = 1.21×10^–36^), transmission of nerve impulse (P_BH_ = 1.69×10^–36^) and neurological system process (P_BH_ = 3.23×10^–32^) were identified (Table 1). Consistently, for the molecular function category, terms related to the activity of neurotransmitter receptor or channel were enriched, such as neurotransmitter receptor activity (P_BH_ = 1.36×10^–26^), excitatory extracellular ligand-gated ion channel activity (P_BH_ = 2.62×10^–22^) and acetylcholine receptor activity (P_BH_ = 5.12×10^–22^). In the cellular component category, the significantly enriched terms included synaptic membrane (P_BH_ = 5.18×10^–27^), neuron projection (P_BH_ = 4.51×10^–24^), axon (P_BH_ = 3.55×10^–21^), dendritic spine (P_BH_ = 9.87×10^–10^). Similarly, GO terms related to drug response (e.g., response to alkaloid, response to nicotine, and response to alcohol) and metabolism (e.g., monooxygenase activity, and oxidoreductase activity), were also enriched in NAGenes. These results were consistent with the pathophysiological background of nicotine addiction, which also indicated the candidate genes are relatively reliable for the following up bioinformatics analysis.

**Table 1:** Gene Ontology terms enriched in nicotine addiction-related genes (NAGenes).

GO Terms^a^		No. of genes^b^	P-value^c^	P_BH_-value^d^
*Biological process*				
GO:0007268	synaptic transmission	67	3.07×10^–39^	1.21×10^–36^
GO:0007267	cell-cell signaling	83	3.85×10^–39^	1.21×10^–36^
GO:0019226	transmission of nerve impulse	70	6.75×10^–39^	1.69×10^–36^
GO:0035637	multicellular organismal signaling	70	2.80×10^–38^	5.86×10^–36^
GO:0050877	neurological system process	82	2.06×10^–34^	3.23×10^–32^
GO:0043279	response to alkaloid	25	2.11×10^–25^	1.89×10^–23^
GO:0014070	response to organic cyclic compound	50	2.74×10^–25^	2.29×10^–23^
GO:0042493	response to drug	41	8.02×10^–25^	6.29×10^–23^
GO:0097305	response to alcohol	23	2.64×10^–21^	1.18×10^–19^
GO:0031644	regulation of neurological system process	30	4.13×10^–21^	1.79×10^–19^
GO:0050890	cognition	27	9.03×10^–21^	3.78×10^–19^
GO:0007611	learning or memory	26	1.36×10^–20^	5.51×10^–19^
GO:0035094	response to nicotine	15	3.61×10^–20^	1.42×10^–18^
GO:0023061	signal release	35	4.76×10^–20^	1.76×10^–18^
GO:0015837	amine transport	21	5.68×10^–20^	2.04×10^–18^
GO:0042417	dopamine metabolic process	14	9.05×10^–20^	3.15×10^–18^
GO:0051952	regulation of amine transport	18	9.50×10^–20^	3.22×10^–18^
GO:0051969	regulation of transmission of nerve impulse	28	1.14×10^–19^	3.76×10^–18^
GO:0050804	regulation of synaptic transmission	27	1.20×10^–19^	3.86×10^–18^
GO:0044057	regulation of system process	39	1.28×10^–19^	4.02×10^–18^
*Molecular function*				
GO:0030594	neurotransmitter receptor activity	25	8.57×10^–29^	1.36×10^–26^
GO:0004889	acetylcholine-activated cation-selective channel activity	14	6.47×10^–25^	5.14×10^–23^
GO:0005230	extracellular ligand-gated ion channel activity	22	4.94×10^–24^	2.62×10^–22^
GO:0005231	excitatory extracellular ligand-gated ion channel activity	19	1.49×10^–23^	5.12×10^–22^
GO:0015464	acetylcholine receptor activity	14	1.61×10^–23^	5.12×10^–22^
GO:0015276	ligand-gated ion channel activity	24	5.08×10^–20^	1.15×10^–18^
GO:0022839	ion gated channel activity	30	1.62×10^–17^	2.58×10^–16^
GO:0038023	signaling receptor activity	60	3.29×10^–17^	4.76×10^–16^
GO:0042166	acetylcholine binding	10	5.34×10^–17^	7.08×10^–16^
GO:0005261	cation channel activity	27	7.74×10^–16^	6.48×10^–15^
GO:0022838	substrate-specific channel activity	32	2.30×10^–15^	1.83×10^–14^
GO:0004888	transmembrane signaling receptor activity	53	3.11×10^–14^	2.06×10^–13^
GO:0035240	dopamine binding	8	4.89×10^–14^	3.11×10^–13^
GO:0015075	ion transmembrane transporter activity	38	9.12×10^–12^	5.58×10^–11^
GO:0008324	cation transmembrane transporter activity	32	9.86×10^–12^	5.81×10^–11^
GO:0022891	substrate-specific transmembrane transporter activity	38	7.70×10^–11^	4.22×10^–10^
GO:0004952	dopamine receptor activity	5	4.67×10^–10^	2.40×10^–9^
GO:0004497	monooxygenase activity	13	4.86×10^–10^	2.41×10^–9^
GO:0016705	oxidoreductase activity, acting on paired donors, withincorporation or reduction of molecular oxygen	16	8.54×10^–10^	4.11×10^–9^
GO:0008227	G-protein coupled amine receptor activity	9	3.52×10^–9^	1.65×10^–8^
GO:0031406	carboxylic acid binding	16	1.16×10^–8^	5.12×10^–8^
GO:0016597	amino acid binding	12	1.69×10^–8^	7.26×10^–8^
GO:0035254	glutamate receptor binding	7	3.26×10^–8^	1.36×10^–7^
GO:0005506	iron ion binding	13	5.60×10^–7^	2.23×10^–6^
*Cellular component*				
GO:0097060	synaptic membrane	35	7.51×10^–29^	5.18×10^–27^
GO:0045211	postsynaptic membrane	32	2.29×10^–27^	1.05×10^–25^
GO:0043005	neuron projection	50	1.96×10^–25^	4.51×10^–24^
GO:0005887	integral to plasma membrane	65	2.72 ×10^–24^	5.36×10^–23^
GO:0031226	intrinsic to plasma membrane	65	1.92×10^–23^	3.31×10^–22^
GO:0005892	acetylcholine-gated channel complex	13	8.13×10^–23^	1.25×10^–21^
GO:0030424	axon	33	2.57×10^–22^	3.55×10^–21^
GO:0043235	receptor complex	25	7.89×10^–20^	9.07×10^–19^
GO:0030054	cell junction	43	2.51×10^–17^	2.48×10^–16^
GO:0030425	dendrite	30	1.58×10^–16^	1.36×10^–15^
GO:0033267	axon part	20	3.63×10^–16^	2.95×10^–15^
GO:0044306	neuron projection terminus	16	1.11×10^–15^	8.51×10^–15^
GO:0043679	axon terminus	15	5.22×10^–15^	3.43×10^–14^
GO:0043025	neuronal cell body	26	5.04×10^–15^	3.43×10^–14^
GO:0043197	dendritic spine	16	1.86×10^–10^	9.87×10^–10^
GO:0044309	neuron spine	16	1.86×10^–10^	9.87×10^–10^
GO:0042734	presynaptic membrane	10	1.54×10^–9^	7.59×10^–9^
GO:0016023	cytoplasmic membrane-bounded vesicle	30	4.81×10^–7^	2.07×10^–6^
GO:0008328	ionotropic glutamate receptor complex	6	1.43×10^–6^	5.80×10^–6^
GO:0044327	dendritic spine head	10	2.19×10^–6^	8.17×10^–6^
GO:0014069	postsynaptic density	10	2.19×10^–6^	8.17×10^–6^

a. Only GO terms with hierarchical level≥4 and containing 5 or more nicotine addiction-related genes are shown.

b. Number of genes in the 220 nicotine addiction-related genes and also in the category

c. P-value were calculated by hypergeometric test

d. P_BH_-value were adjusted by Benjamini & Hochberg (BH) method

### Pathway enrichment analysis in NAGenes

Identifying biological pathways enriched in the candidate genes may provide important information for our understanding of the molecular mechanism underlying nicotine addiction. We searched for enriched pathways in the NAGenes using IPA and found 97 significant enrichment pathways (P_BH_≤0.01) (S2 Table). The 20 most significantly enrichment pathways are shown in Table 2. Most of the pathways were related to neurotransmission system, consistent with the fact that nicotine addiction is a neuronal disease. Among them, several pathways associated with monoamine neurotransmitters stood out, e.g., dopamine receptor signaling (ranked 4^th^), serotonin receptor signaling (ranked 11^th^), glutamate receptor signaling (ranked 14^th^) and GABA receptor signaling(ranked 19^th^), all of which play important roles in signaling transduction. Moreover, two pathways, synaptic long term potentiation (P_BH_ = 1.07×10^–3^) and synaptic long term depression (P_BH_ = 8.13×10–3) were enriched in the NAGenes (S2 Table). These two pathways were critical in synaptic plasticity development and have been reported to be involved in addiction [26–27]. In addition, we also highlighted other significantly enriched pathways, i.e., cAMP-mediated signaling, calcium signaling, G-protein coupled receptor signaling, neuropathic pain signaling in dorsal horn neurons and CREB signaling in neurons. This result was consistent with prior knowledge of nicotine addiction [28–30], providing valuable evidence for the study of molecular mechanism underlying nicotine addiction. Of note, we found many pathways that were related to immune system in the list, in line with previous reports that nicotine might have effects on organism’s immune [2, 31]. Moreover, three pathways related to retinoid X receptor (RXR) were found to be enriched in the NAGenes, i.e., LPS/IL-1 mediated inhibition of RXR function, PXR (pregnane X receptor)/RXR activation and LXR (liver X receptor)/RXR activation. RXR, a kind of nuclear receptor, is a master regulator during ligand-induced transcription activities [32]. In summary, these results suggest that neurodevelopment, immune and metabolic systems play important roles in the pathogenesis of nicotine addiction.

**Table 2:** Pathways enriched in nicotine addiction-related genes (NAGenes) (top 20 pathways).

Canonical Pathways	P-value^a^	P_BH_-value^b^	NAGenes included in the pathway^c^
cAMP-mediated signaling	6.31×10^–17^	2.00×10^–14^	ADRA2A, ADRB2, AGTR1, AKAP13, CAMK4, CHRM1, CHRM2, CHRM5, CNR1, CREB1, DRD1, DRD2, DRD3, DRD4, DRD5, GABBR1, GABBR2, GNAS, GRM7, HTR1F, HTR6, NPY1R, OPRM1, PDE4D, RAPGEF3
Calcium Signaling	1.00×10^–15^	2.00×10^–13^	CAMK4, CHRNA1, CHRNA10, CHRNA2, CHRNA3, CHRNA4, CHRNA5, CHRNA6, CHRNA7, CHRNB1, CHRNB2, CHRNB3, CHRNB4, CHRND, CHRNG, CREB1, GRIK1, GRIN2A, GRIN2B, GRIN3A, ITPR2, TRPC7
G-Protein Coupled Receptor Signaling	2.51×10^–15^	3.16×10^–13^	ADRA2A, ADRB2, AGTR1, CAMK4, CHRM1, CHRM2, CHRM5, CNR1, CREB1, DRD1, DRD2, DRD3, DRD4, DRD5, GABBR1, GABBR2, GNAS, GRM7, HTR1F, HTR2A, HTR6, NPY1R, OPRM1, PDE4D, RAPGEF3
Dopamine Receptor Signaling	3.16×10^–14^	2.51×10^–12^	COMT, DRD1, DRD2, DRD3, DRD4, DRD5, GNAS, MAOA, MAOB, NCS1, PPP1R1B, PPP2R2B, SLC18A2, SLC6A3, TH
Xenobiotic Metabolism Signaling	2.00×10^–12^	1.41×10^–10^	ABCB1, AHR, CAMK4, CYP1A1, CYP2B6, FMO1, GSTM1, GSTM3, GSTP1, GSTT1, IL6, MAOA, MAOB, MAP3K4, MGMT, NOS2, NQO1, PPP2R2B, SOD3, SULT1A1, TNF, UGT1A9, UGT2B10
Dopamine-DARPP32 Feedback in cAMP Signaling	2.69×10^–10^	1.45×10^–8^	CAMK4, CREB1, DRD1, DRD2, DRD3GRIN3A, DRD4, DRD5, GNAS, GRIN2A, GRIN2B, ITPR2, KCNJ6, PPP1R1B, PPP2R2B, PRKG1
Aryl Hydrocarbon Receptor Signaling	2.88×10^–10^	1.45×10^–8^	AHR, CCND1, CHEK2, CYP1A1, ESR1, GSTM1, GSTM3, GSTP1, GSTT1, IL6, MDM2, NQO1, TGFB1, TNF, TP53
LPS/IL-1 Mediated Inhibition of RXR Function	4.37×10^–10^	1.78×10^–8^	ABCB1, ABCC4, APOE, CD14, CETP, CYP2A6, CYP2B6, FMO1, GSTM1, GSTM3, GSTP1, GSTT1, MAOA, MAOB, MGMT, SOD3, SULT1A1, TNF
Gαi Signaling	4.57×10^–10^	1.78×10^–8^	ADRA2A, AGTR1, CHRM2, CNR1, DRD2, DRD3, DRD4, GABBR1, GABBR2, GNAS, GRM7, HTR1F, NPY1R, OPRM1
Superpathway of Melatonin Degradation	3.72×10^–9^	1.32×10^–7^	CYP1A1, CYP2A6, CYP2B6, CYP2D6, MAOA, MAOB, MPO, SULT1A1, UGT1A9, UGT2B10
Serotonin Receptor Signaling	6.17×10^–9^	1.95×10^–7^	HTR2A, HTR6, MAOA, MAOB, SLC18A2, SLC6A4, TPH1, TPH2
eNOS Signaling	1.17×10^–8^	3.39×10^–7^	CAMK4, CHRNA10, CHRNA3, CHRNA4, CHRNA5, CHRNB1, CHRNB4, ESR1, GNAS, HSPA4, ITPR2, NOS3, PRKG1
Glucocorticoid Receptor Signaling	3.89×10^–8^	1.05×10^–6^	ADRB2, CCNH, CREB1, ERCC2, ESR1, HSPA4, ICAM1, IFNG, IL13, IL6, IL8, NOS2, NPPA, NR3C1, PTGS2, TGFB1, TNF
Glutamate Receptor Signaling	5.13×10^–8^	1.29×10^–6^	CAMK4, DLG4, GRIK1, GRIK2, GRIN2A, GRIN2B, GRIN3A, GRM7, SLC1A2
Neuropathic Pain Signaling In Dorsal Horn Neurons	5.50×10^–7^	1.29×10^–5^	BDNF, CAMK4, CREB1, GRIN2A, GRIN2B, GRIN3A, GRM7, ITPR2, KCNQ3, NTRK2
AMPK Signaling	1.10×10^–6^	2.40×10^–5^	ADRA2A, ADRB2, CHRNA10, CHRNA3, CHRNA4, CHRNA5, CHRNB1, CHRNB4, GNAS, NOS3, PPP2R2B
Hepatic Cholestasis	1.58×10^–6^	3.09×10^–5^	ABCB1, CD14, CETP, ESR1, GNAS, IFNG, IL6, IL8, MAP3K4, SLCO3A1, TNF
Gαs Signaling	1.58×10^–6^	3.09×10^–5^	ADRB2, CHRM1, CHRM5, CNR1, CREB1, DRD1, DRD5, GNAS, HTR6, RAPGEF3
GABA Receptor Signaling	1.95×10^–6^	3.47×10^–5^	DNM1, GABARAP, GABBR1, GABBR2, GABRA2, GABRA4, GABRE
PXR/RXR Activation	2.00×10^–6^	3.47×10^–5^	ABCB1, CYP2A6, CYP2B6, GSTM1, IL6, NR3C1, TNF, UGT1A9

^a^ P-value were calculated by Fisher’s exact test

^b^ P_BH_-value were adjusted by Benjamini & Hochberg (BH) method

^c^ 220 nicotine addiction-related genes included in the pathway

### Crosstalk among significantly enriched pathway

**Fig 1:**
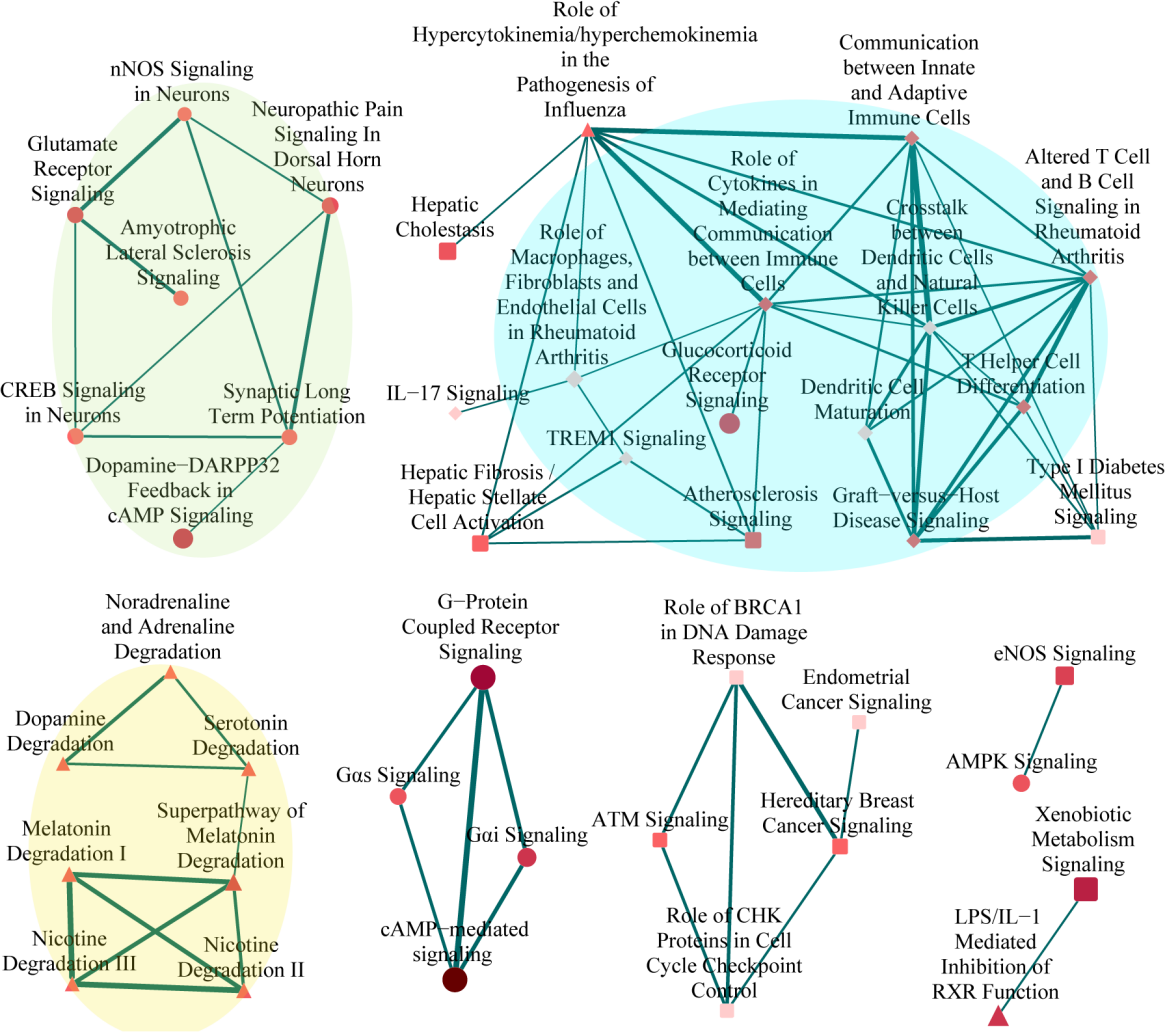
Pathway crosstalk among NAGenes-enriched pathways. Nodes represent pathways and edges represent crosstalk between pathways. Node size corresponds to the number of NAGenes found in the corresponding pathway. Node color corresponds to the P_BH_-value of the corresponding pathway. Darker color indicates lower P_BH_-value. Edge width corresponds to the score of the related pathways. Node shape indicates pathway categories, with ellipse for neurodevelopment, diamond for immune, triangle for metabolism, square for other pathways.

To take a further step beyond identifying lists of significantly enriched pathways and to understand how they interact with each other, we performed a pathway crosstalk analysis among the 97 significantly enriched pathways. The approach was based on the assumption that two pathways were considered to crosstalk if they shared a proportion of NAGenes [16]. There were 74 pathways containing 5 or more members in NAGenes, of which, 72 pathways met the criterion for crosstalk analysis, i.e., each pathway shared at least 3 genes with one or more other pathways. There were a total of 380 pathway pairs (edges) from the 72 pathways and then we ranked these edges according to the average scores of the JC and the OC. Ultimately, we chose the top 20% edges to construct the pathway crosstalk. Based on their crosstalk, the pathways could be roughly grouped into three major modules, each of which included pathways shared more crosstalks compared with other pathways and may likely be involved in the same or similar biological process (Fig 1). One module mainly consisted of neurodevelopment-related signaling pathways, such as glutamate receptor signaling, synaptic long term potentiation and CREB signaling in neurons. The second module was primarily dominated by immune system-related pathways, including role of cytokines in mediating communication between immune cells, T helper cell differentiation and others. Another module composed of the metabolic pathways of neurotransmitters or drug, such as nicotine degradation II, dopamine degradation and serotonin degradation, the roles of these pathways in nicotine addiction have not been fully explored. As indicated by the above results, pathway crosstalk analysis can provide important insights for understanding of nicotine addiction mechanisms.

### Nicotine addiction-specific network

**Fig 2:**
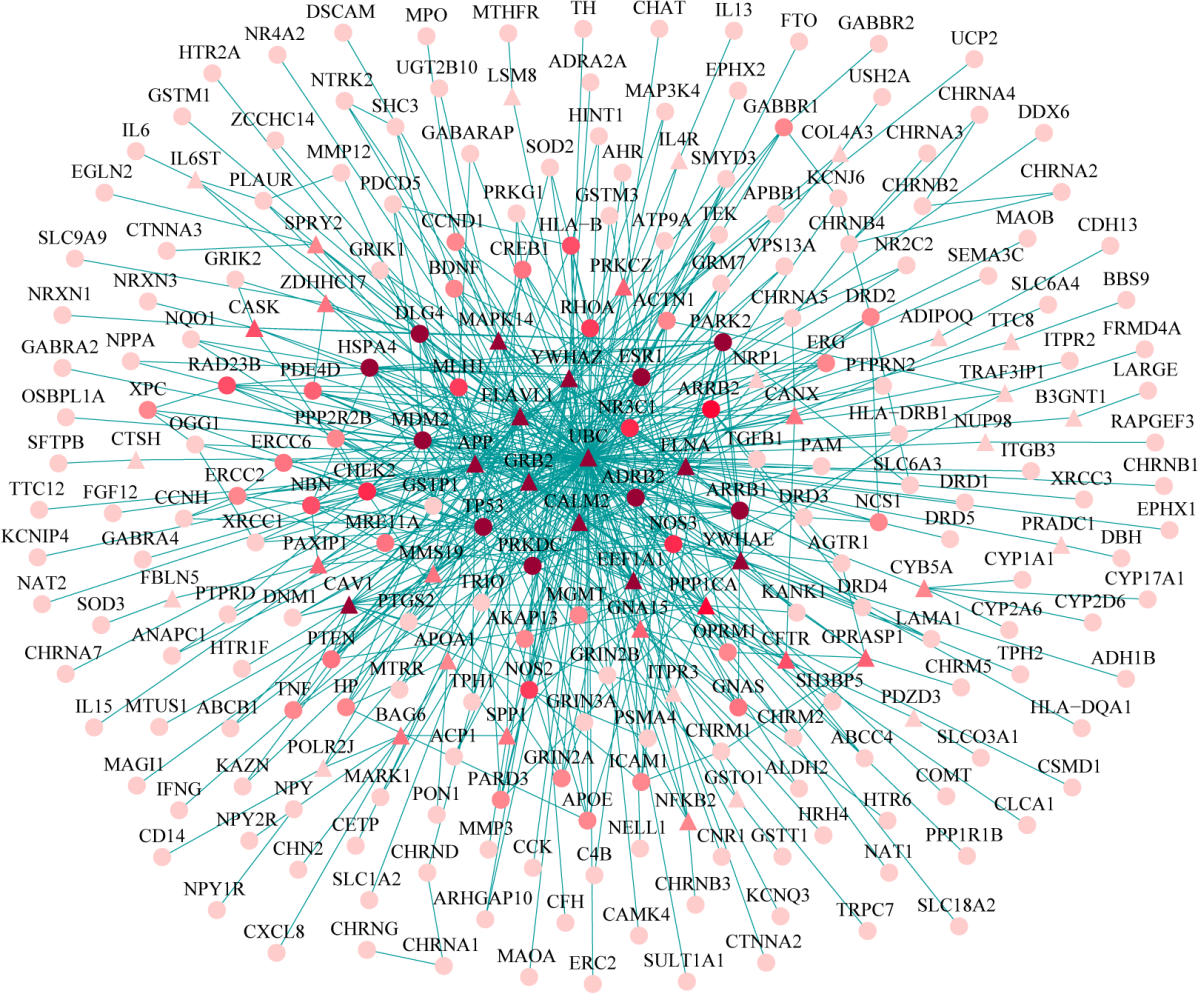
Nicotine addiction-specific network. Ellipse nodes are NAGenes and triangular nodes are non-NAGenes. Node color corresponds to its degree in the human PPI network. Darker color indicates higher degree.

To distill insight into the interaction of NAGenes in a local environment, we extracted the specific subnetwork of nicotine addiction (NA-specific network) from the human PPI network using the Steiner minimal tree algorithm. Basically, this approach linked as many as possible members of NAGenes via the minimal number of connections. As shown in Fig 2, the subnetwork contained 252 nodes and 591 edges. Of the 220 NAGenes, 208 were included in the NA-specific network, which accounted for approximately 94.5% of the candidate genes and 82.5% of the genes in the NA-specific network, indicating a high coverage of NAGenes in the subnetwork. Of note, some of the 44 additional genes, e.g., calmodulin 2 (CALM2), calnexin (CANX), caveolin-1 (CAV1), glutathione S-transferase omega 1 (GSTO1) and protein phosphatase 1 (PPP1CA), had been reported to be associated with addiction in previously studies (Table 3) [33–34].

**Table 3:** Genes included in the NA-specific network but not of NAGenes.

Gene symbol	Gene name
ADIPOQ	Adiponectin
APOA1	Apolipoprotein A-I
APP	Amyloid beta (A4) precursor protein
B3GNT1	UDP-GlcNAc:betaGal beta-1,3-N-acetylglucosaminyltransferase 1
BAG6	BCL2-associated athanogene 6
CALM2	Calmodulin 2
CANX	Calnexin
CASK	Calcium/calmodulin-dependent serine protein kinase
CAV1	Caveolin 1
CFTR	Cystic fibrosis transmembrane conductance regulator
COL4A3	Collagen, type IV, alpha 3
CTSH	Cathepsin H
CYB5A	Cytochrome b5 type A
EEF1A1	Eukaryotic translation elongation factor 1 alpha 1
ELAVL1	ELAV like RNA binding protein 1
FBLN5	Fibulin 5
FLNA	Filamin A, alpha
GNA15	Guanine nucleotide binding protein (G protein), alpha 15 (Gq class)
GPRASP1	G protein-coupled receptor associated sorting protein 1
GRB2	Growth factor receptor-bound protein 2
GSTO1	Glutathione S-transferase omega 1
IL4R	Interleukin 4 receptor
IL6ST	Interleukin 6 signal transducer
ITPR3	Inositol 1,4,5-trisphosphate receptor, type 3
LSM8	LSM8 homolog, U6 small nuclear RNA associated
MAPK14	Mitogen-activated protein kinase 14
MMS19	MMS19 nucleotide excision repair homolog
NFKB2	Nuclear factor of kappa light polypeptide gene enhancer in B-cells 2
NRP1	Neuropilin 1
NUP98	Nucleoporin 98kDa
PAXIP1	PAX interacting (with transcription-activation domain) protein 1
PDZD3	PDZ domain containing 3
POLR2J	Polymerase (RNA) II (DNA directed) polypeptide J
PPP1CA	Protein phosphatase 1, catalytic subunit,alpha isozyme
PRADC1	Protease-associated domain containing 1
PRKCZ	Protein kinase C, zeta
SPP1	Secreted phosphoprotein 1
SPRY2	Sprouty homolog 2
TRAF3IP1	TNF receptor-associated factor 3 interacting protein 1
TTC8	Tetratricopeptide repeat domain 8
UBC	Ubiquitin C
YWHAE	Tyrosine 3-monooxygenase/tryptophan 5-monooxygenase activation protein, epsilon
YWHAZ	Tyrosine 3-monooxygenase/tryptophan 5-monooxygenase activation protein, zeta
ZDHHC17	Zinc finger, DHHC-type containing 17

Moreover, to test randomness of the NA-specific network, 1000 random subnetworks were generated using the Erdos-Renyi model and their average shortest-path distance and average clustering coefficient were compared with the corresponding values of the NA-specific network. For these random subnetworks, the average shortest-path distance was 3.72, which was significantly larger than that of the NA-specific network (shortest-path distance, 2.87; empirical p value < 0.001). The average clustering coefficient of the random subnetworks was 0.02, which was significantly smaller than that of the NA-specific network (clustering coefficient, 0.25; empirical p value < 0.001). Thus, the NA-specific network extracted from the whole PPI network was a non-random network.

### Molecular network of nicotine addiction

**Fig 3:**
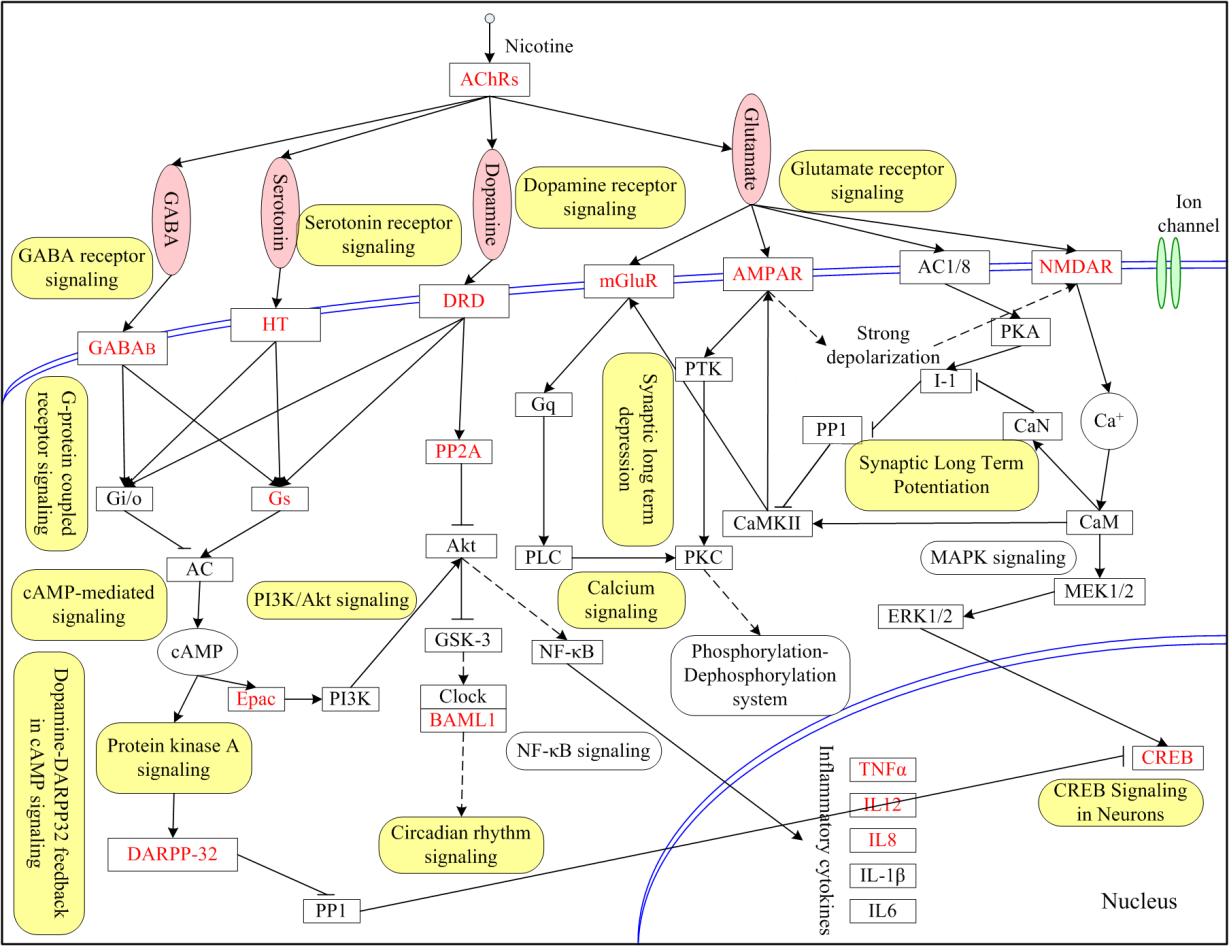
Molecular network for nicotine addiction. This network is constructed based on the nicotine addiction-related pathways or pathway crosstalk identified in our study, NA-specific network and literature survey. NAGenes are highlighted in red while neurotransmitters are represented as pink ellipse. The enriched pathways are highlighted in yellow background.

Summarizing the results from pathway analysis and network analysis, we were able to obtain a relatively comprehensive view about nicotine addiction (Fig 3). In such network, a number of key genes and pathways played important roles, e.g., the neurotransmitters receptor signaling transduction pathways such as dopamine receptor signaling and serotonin receptor signaling, and several intracellular signaling transduction cascades such as cAMP-mediated signaling, G-protein coupled receptor signaling and calcium signaling. The network also included several feedback loops, among which the one from N-methyl-D-aspartate subtype glutamate receptor (NMDAR) to a-amino-3-hydroxyl- 5-methylisoxazole-4-propionic acid subtype glutamate receptor (AMPAR) would be considered the shortest loops. We further observed that some loops interlinked with each other through CaM (also called CALM) and calcium/calmodulin-dependent protein kinase II (CAMKII). CaM and CAMKII both play important roles in the induction of long term potentiation and long term depression, indicating that they might make contribution to the synaptic plasticity development and they might provide clues to explain the irreversible features of nicotine addiction. In addition, we observed that mitogen-activated protein kinase (MAPK), nuclear factor of kappa light polypeptide gene enhancer in B-cells (NF-κB) and related pathways of NF-κB signaling and MAPK signaling also implicated in the process of nicotine addiction, which might be the valuable candidate genes or pathways.

## Discussion

In the past decades, we have made considerable progress in understanding the molecular mechanisms underlying nicotine addiction, which is largely due to the identification of various neurotransmitter receptors, genes or pathways associated with addiction and the development of animal or cell models. Additionally, with the development of high throughput analysis technology, more and more genes/proteins have been suggested to be linked to nicotine addiction and provide a valuable resource to analyze candidate genes function, biochemical pathways and networks related to nicotine addiction. In this study, we provided a comprehensive analysis of the functional features and interaction network of nicotine addiction-related genes.

Function enrichment analysis revealed the specific biological processes involved by NAGenes. Our GO enrichment analysis indicated that these genes participated in neurodevelopment-related process and ion channel or neurotransmitters activity. For example, terms such as acetylcholine receptor activity, dopamine receptor activity and glutamate receptor binding were significantly enriched in NAGenes, indicating the importance of these neurotransmitters in the development of nicotine addiction. Of note, we found the GO terms of cognition and learning or memory were also in the enriched list, consistent with previous findings of the roles of nicotine in the regulation of various physiological processes, including learning and memory [35–36].

Pathway analysis revealed that pathways related to neurodevelopment were enriched in NAGenes, which further verified the existence of close relationship between the pathology of nicotine addiction and the signaling pathways of nervous system. Four pathways that were related to monoamine neurotransmitters were found to be enriched in the NAGenes, consistent with their central roles in the development of nicotine addiction. Stimulation of nicotinic acetylcholine receptors (nAChR) releases a variety of neurotransmitters in the brain, e.g., dopamine, serotonin, glutamate and GABA. Dopamine is critical for the reinforcing effects or rewarding behaviors of nicotine [37–38], glutamate and GABA are respectively major excitatory and inhibitory neurotransmitter and both of them play important roles in the development of nicotine addiction [39–40]. These neurotransmitters interact with the specific receptors, triggering a series of neuronal signaling pathways and then ultimately realize the regulation of various physiological processes. Of note, our analysis indicated that two pathways, synaptic long term potentiation and synaptic long term depression, were also enriched in genes associated with nicotine addiction. Repeated stimulation of nicotine to nervous system ultimately can modify the neural circuitry and thereby lead to addiction. As for many forms of experience-dependent synaptic plasticity, synaptic long term potentiation and synaptic long term depression play critical roles in the formation, maintenance, and appropriate functioning of neural circuits [41–42]. Therefore, these two pathways might be involved in the early stages of the development of nicotine addiction and facilitate the adaptation of body to changing environments. In addition, synaptic plasticity, as the molecular basis of learning and memory in the nervous system, has been extensively studied. Synaptic long term potentiation and synaptic long term depression have been reported to underline the cognitive and memory effects of the addictive potential of some drugs of abuse [43–44]. This further proved that nicotine could directly or indirectly modulate the physiological processes of learning and memory. Moreover, three signal pathways related to RXR were identified, i.e., LPS/IL-1 mediated inhibition of RXR function, PXR/RXR activation and LXR/RXR activation. Retinoic acid (RA), a class of natural or synthetic vitamin A analogs, exert profound effects on many biological processes, such as development, differentiation and maintenance of nervous system [45] and have been reported that it may serve as potential bridge between the genetic and environmental components of complex diseases [46–47], suggesting that environmental factors also play an important role in nicotine addiction. Interestingly, we found the circadian rhythm signaling was also in the enriched pathway list, supporting that there might be a link between nicotine addiction and abnormal or disrupted circadian rhythms [48]. As indicated by these results, the molecular mechanisms underlying nicotine addiction are quite complex and involve many genes, pathways and their interactions.

Of significance, in pathway crosstalk analysis we identified three main modules. One module was mainly dominated by the pathways associated with the activity of the nervous system. Among these pathways, dopamine-DARPP32 feedback in cAMP signaling, glutamate receptor signaling, neuropathic pain signaling in dorsal horn neurons, and CREB signaling in neurons have been well studied to be involved in neuron or central nervous system (CNS) [49–51]. For instance, CREBs, widely been accepted as prototypical transcription factors, play a critical role in biological processes such as neuronal plasticity, learning and memory. Meanwhile, several lines of evidence have pointed that alterations of the activity of CREB by drugs of abuse have a profound effect on behavioral manifestations of drug reward and withdrawal [52]. Subsequently, we collected the genes contributing to the crosstalk, and the most frequently shared genes included glutamate receptor ionotropic N-methyl D-aspartate 2A (GRIN2A), GRIN2B, GRIN3A, calcium/calmodulin-dependent protein kinase IV (CAMK4), CREB1, and glutamate receptor metabotropic 7 (GRM7), suggesting these genes might be more potential targets in the development of nicotine addiction. In addition, the pathway pair of cAMP-mediated signaling and G-protein coupled receptor signaling, which were not included in the three modules, was also noteworthy. In the pathway analysis, we found these two pathways stood out at the top of the list by the statistically significant level (P_BH_ = 2.00×10^–14^, ranked 1^st^ and P_BH_ = 3.16×10^–13^, ranked 3^rd^, respectively) (S2 Table). And the score of this pathway pair was 0.94, ranking the first. Furthermore, these two pathways have been deeply studied for their functions in the nervous system, such as regulating pivotal physiological processes. It was worth noting that, several edges, linking any one of these two pathways and other significant pathways were not displayed in Fig 1 just because they did not meet our criteria, such as the link between cAMP-mediated signaling and dopamine receptor signaling. In this study, we just empirically chose the pathway pairs whose crosstalk score fell within the top 20%, therefore some pathway pairs which we might be interested in were not shown in Fig 1.

We further extracted the NA-specific network from the reference network. It was interesting to note that some additional genes not among the NAGenes but included in the human PPI network, were associated with nicotine addiction. For instance, CAV1, the structural protein of caveolae, can regulate the function of dopamine receptor D1 in glial cells and hippocampal neurons and may participate in G protein-coupled receptor signaling events [53–54]. CANX, a transmembrane protein in endoplasmic reticulum, can mediate intracellular Ca^2+^ concentration and directly interact with dopamine and G protein-coupled receptors to regulate their expression (Table 3) [55–56]. As indicated by the results, network-based analysis could not only provide meaningful information about the organization and environment of NAGenes, but also be promising to identify novel candidate genes. Although the quantity and quality of PPI data have been greatly improved, the human PPI network is still far from complete. In such scenario, some proteins may simply have more interaction information than others because they are better studied, instead of they are biologically more important. Also, due to the limitation of current technology, there may be some false positives in the PPI data [57]. Such potential biases associated with human PPI network may affect our interpretation of the results.

## Conclusions

The neurobiological processes that underlie nicotine addiction are complex which relate to multiple factors, such as genetic and environmental factors. In this study, we applied a systems biology framework for a comprehensive functional analysis of nicotine addiction using candidate genes prioritized via a multi-source-based approach. Through integrating the information from GO, pathway and pathway crosstalk analysis, we found neurotransmitters or neurodevelopment-related signal pathway and immune system play key roles in the molecular mechanism of nicotine addiction. Further, we extracted nicotine addiction-specific subnetwork, in which some of the additional genes had been reported to be involved in nicotine addiction. To distill the global view of nicotine addiction process, we preliminarily constructed a molecular network for it. Our results provide important information for the further analysis and suggest that system level analysis is promising for understanding the pathophysiology of nicotine addiction.

## Supporting Information

S1 Table List of the 220 Nicotine addiction-related genes (NAGenes). (DOC)

S2 Table Pathways significantly enriched in NAGenes. (DOC)
