# Supplementary material for: Pathways and Networks-Based Analysis of Candidate Genes Associated with Nicotine Addiction
Source: PLoS One. 2015 May 12;10(5):e0127438. doi: 10.1371/journal.pone.0127438 (PMC4429103; doi:10.1371/journal.pone.0127438)
Supplement: S1 Table — (DOC) [file pone.0127438.s001.doc]

**S1 Table. List of the 220 Nicotine addiction-related genes (NAGenes).**

| **Gene Symbol** | **Gene Name** |
| --- | --- |
| ADRB2 | Adrenergic, beta 2, receptor, surface |
| BDNF | Brain-derived neurotrophic factor |
| CCND1 | Cyclin D1 |
| CCNH | Cyclin H |
| CHRNA10 | Cholinergic receptor, nicotinic, alpha 10 |
| CHRND | Cholinergic receptor, nicotinic, delta |
| CHRNG | Cholinergic receptor, nicotinic, gamma |
| CYP17A1 | Cytochrome P450, family 17, subfamily A, polypeptide 1 |
| DRD4 | Dopamine receptor D4 |
| FGF12 | Fibroblast growth factor 12 |
| GABRA2 | Gamma-aminobutyric acid (GABA) A receptor, alpha 2 |
| GNAS | GNAS complex locus |
| GSTP1 | Glutathione S-transferase pi 1 |
| ITPR2 | Inositol 1,4,5-triphosphate receptor, type 2 |
| LARGE | Like-glycosyltransferase |
| NRXN1 | Neurexin 1 |
| PPP2R2B | Protein phosphatase 2 (formerly 2A), regulatory subunit B, beta isoform |
| PTEN | Phosphatase and tensin homolog; phosphatase and tensin homolog pseudogene 1 |
| PTPRD | Protein tyrosine phosphatase, receptor type, D |
| SFTPB | Surfactant protein B |
| SHC3 | SHC (Src homology 2 domain containing) transforming protein 3 |
| SLC18A2 | Solute carrier family 18 (vesicular monoamine), member 2 |
| SLC6A4 | Solute carrier family 6 (neurotransmitter transporter, serotonin), member 4 |
| SOD2 | Superoxide dismutase 2, mitochondrial |
| SULT1A1 | Sulfotransferase family, cytosolic, 1A, phenol-preferring, member 1 |
| TH | Tyrosine hydroxylase |
| ABCC4 | ATP-binding cassette, sub-family C (CFTR/MRP), member 4 |
| ACTN1 | Actinin, alpha 1 |
| ADH1B | Alcohol dehydrogenase 1B (class I), beta polypeptide |
| ALDH2 | Aldehyde dehydrogenase 2 family (mitochondrial) |
| CCK | Cholecystokinin |
| CDH13 | Cadherin 13, H-cadherin (heart) |
| CHRNA1 | Cholinergic receptor, nicotinic, alpha 1 |
| CHRNA2 | Cholinergic receptor, nicotinic, alpha 2 |
| CHRNA3 | Cholinergic receptor, nicotinic, alpha 3 |
| CHRNA4 | Cholinergic receptor, nicotinic, alpha 4 |
| CHRNA5 | Cholinergic receptor, nicotinic, alpha 5 |
| CHRNA6 | Cholinergic receptor, nicotinic, alpha 6 |
| CHRNA7 | Cholinergic receptor, nicotinic, alpha 7 |
| CHRNB1 | Cholinergic receptor, nicotinic, beta 1 |
| CHRNB2 | Cholinergic receptor, nicotinic, beta 2 |
| CHRNB3 | Cholinergic receptor, nicotinic, beta 3 |
| CHRNB4 | Cholinergic receptor, nicotinic, beta 4 |
| CNR1 | Cannabinoid receptor 1 (brain) |
| CREB1 | Camp responsive element binding protein 1 |
| CYP1A1 | Cytochrome P450, family 1, subfamily A, polypeptide 1 |
| DDX6 | DEAD (Asp-Glu-Ala-Asp) box polypeptide 6 |
| DNM1 | Dynamin 1 |
| GABARAP | GABA(A) receptor-associated protein |
| GABBR1 | Gamma-aminobutyric acid (GABA) B receptor, 1 |
| GRIK1 | Glutamate receptor, ionotropic, kainate 1 |
| GRIK2 | Glutamate receptor, ionotropic, kainate 2 |
| HLA-DRB1 | Major histocompatibility complex, class II, DR beta 1 |
| HSPA4 | Heat shock 70kda protein 4 |
| HTR2A | 5-hydroxytryptamine (serotonin) receptor 2A |
| IL15 | Interleukin 15 |
| IL6 | Interleukin 6 (interferon, beta 2) |
| IL8 | Interleukin 8 |
| LAMA1 | Laminin, alpha 1 |
| MAOB | Monoamine oxidase B |
| MRE11A | MRE11 meiotic recombination 11 homolog A (S. Cerevisiae) |
| NPY | Neuropeptide Y |
| NQO1 | NAD(P)H dehydrogenase, quinone 1 |
| NR2C2 | Nuclear receptor subfamily 2, group C, member 2 |
| NTRK2 | Neurotrophic tyrosine kinase, receptor, type 2 |
| OGG1 | 8-oxoguanine DNA glycosylase |
| PTGS2 | Prostaglandin-endoperoxide synthase 2 |
| RAD23B | RAD23 homolog B (S. Cerevisiae) |
| RAPGEF3 | Rap guanine nucleotide exchange factor (GEF) 3 |
| RHOA | Ras homolog gene family, member A |
| SEMA3C | Sema domain, immunoglobulin domain (Ig), short basic domain, secreted, (semaphorin) 3C |
| SLC9A9 | Solute carrier family 9 (sodium/hydrogen exchanger), member 9 |
| TGFB1 | Transforming growth factor, beta 1 |
| TNF | Tumor necrosis factor (TNF superfamily, member 2) |
| TP53 | Tumor protein p53 |
| ADRA2A | Adrenergic, alpha-2A-, receptor |
| ANAPC1 | Anaphase promoting complex subunit 1; |
| APBB1 | Amyloid beta (A4) precursor protein-binding, family B, member 1 |
| ATP9A | Atpase, class II, type 9A |
| CD14 | CD14 molecule |
| CHAT | Choline acetyltransferase |
| CHEK2 | Protein kinase CHK2-like; CHK2 checkpoint homolog (S. Pombe) |
| CHRM1 | Cholinergic receptor, muscarinic 1 |
| CHRM2 | Cholinergic receptor, muscarinic 2 |
| COMT | Catechol-O-methyltransferase |
| CTNNA2 | Catenin (cadherin-associated protein), alpha 2 |
| CTNNA3 | Catenin (cadherin-associated protein), alpha 3 |
| CYP2D6 | Cytochrome P450, family 2, subfamily D, polypeptide 6 |
| DRD1 | Dopamine receptor D1 |
| ERCC6 | Excision repair cross-complementing rodent repair deficiency, complementation group 6 |
| ESR1 | Estrogen receptor 1 |
| FTO | Fat mass and obesity associated |
| GABBR2 | Gamma-aminobutyric acid (GABA) B receptor, 2 |
| GABRA4 | Gamma-aminobutyric acid (GABA) A receptor, alpha 4 |
| GRIN2A | Glutamate receptor, ionotropic, N-methyl D-aspartate 2A |
| GRIN2B | Glutamate receptor, ionotropic, N-methyl D-aspartate 2B |
| GSTT1 | Glutathione S-transferase theta 1 |
| ITGB3 | Integrin, beta 3 (platelet glycoprotein iiia, antigen CD61) |
| KCNIP4 | Kv channel interacting protein 4 |
| MAP3K4 | Mitogen-activated protein kinase kinase kinase 4 |
| MPO | Myeloperoxidase |
| NELL1 | NEL-like 1 (chicken) |
| NOS2 | Nitric oxide synthase 2, inducible |
| NOS3 | Nitric oxide synthase 3 (endothelial cell) |
| NR3C1 | Nuclear receptor subfamily 3, group C, member 1 (glucocorticoid receptor) |
| OPRM1 | Opioid receptor, mu 1 |
| PAM | Peptidylglycine alpha-amidating monooxygenase |
| PARK2 | Parkinson disease (autosomal recessive, juvenile) 2, parkin |
| PPP1R1B | Protein phosphatase 1, regulatory (inhibitor) subunit 1B |
| PRKG1 | Protein kinase, cgmp-dependent, type I |
| SLC1A2 | Solute carrier family 1 (glial high affinity glutamate transporter), member 2 |
| SOD3 | Superoxide dismutase 3, extracellular |
| TAS2R38 | Taste receptor, type 2, member 38 |
| TEK | TEK tyrosine kinase, endothelial |
| TPH1 | Tryptophan hydroxylase 1 |
| UGT1A7 | UDP glucuronosyltransferase 1 family, polypeptide A7; |
| ABCB1 | ATP-binding cassette, sub-family B (MDR/TAP), member 1 |
| ACP1 | Acid phosphatase 1, soluble |
| AGTR1 | Angiotensin II receptor, type 1 |
| AHR | Aryl hydrocarbon receptor |
| AKAP13 | A kinase (PRKA) anchor protein 13 |
| ANKK1 | Ankyrin repeat and kinase domain containing 1 |
| APOE | Apolipoprotein E |
| ARHGAP10 | Rho gtpase activating protein 10 |
| ARRB1 | Arrestin, beta 1 |
| ARRB2 | Arrestin, beta 2 |
| BBS9 | Bardet-Biedl syndrome 9 |
| C4B | Complement component 4B (Chido blood group) |
| CAMK4 | Calcium/calmodulin-dependent protein kinase IV |
| CETP | Cholesteryl ester transfer protein, plasma |
| CFH | Complement factor H |
| CHN2 | Chimerin (chimaerin) 2 |
| CHRM5 | Cholinergic receptor, muscarinic 5 |
| CLCA1 | Chloride channel accessory 1 |
| CLSTN2 | Calsyntenin 2 |
| CSMD1 | CUB and Sushi multiple domains 1 |
| CYP2A6 | Cytochrome P450, family 2, subfamily A, polypeptide 6 |
| CYP2B6 | Cytochrome P450, family 2, subfamily B, polypeptide 6 |
| DBH | Dopamine beta-hydroxylase (dopamine beta-monooxygenase) |
| DLG4 | Discs, large homolog 4 (Drosophila) |
| DRD2 | Dopamine receptor D2 |
| DRD3 | Dopamine receptor D3 |
| DRD5 | Dopamine receptor D5 |
| DSCAM | Down syndrome cell adhesion molecule |
| EGLN2 | Egl nine homolog 2 (C. Elegans) |
| EPHX1 | Epoxide hydrolase 1, microsomal (xenobiotic) |
| EPHX2 | Epoxide hydrolase 2, cytoplasmic |
| ERC2 | ELKS/RAB6-interacting/CAST family member 2 |
| ERCC2 | Excision repair cross-complementing rodent repair deficiency, complementation group 2 |
| ERG | V-ets erythroblastosis virus E26 oncogene homolog (avian) |
| FMO1 | Flavin containing monooxygenase 1 |
| FRMD4A | FERM domain containing 4A |
| GABRE | Gamma-aminobutyric acid (GABA) A receptor, epsilon |
| GALR1 | Galanin receptor 1 |
| GRIN3A | Glutamate receptor, ionotropic, N-methyl-D-aspartate 3A |
| GRM7 | Glutamate receptor, metabotropic 7 |
| GSTM1 | Glutathione S-transferase mu 1 |
| GSTM3 | Glutathione S-transferase mu 3 (brain) |
| HINT1 | Histidine triad nucleotide binding protein 1 |
| HLA-B | Major histocompatibility complex, class I, C; major histocompatibility complex, class I, B |
| HLA-DQA1 | Major histocompatibility complex, class II, DQ alpha 1 |
| HP | Haptoglobin-related protein; haptoglobin |
| HRH4 | Histamine receptor H4 |
| HTR1F | 5-hydroxytryptamine (serotonin) receptor 1F |
| HTR6 | 5-hydroxytryptamine (serotonin) receptor 6 |
| ICAM1 | Intercellular adhesion molecule 1 |
| IFNG | Interferon, gamma |
| IL13 | Interleukin 13 |
| KANK1 | KN motif and ankyrin repeat domains 1; similar to ankyrin repeat domain protein 15 isoform b |
| KAZN | Kazrin, periplakin interacting protein |
| KCNJ6 | Potassium inwardly-rectifying channel, subfamily J, member 6 |
| KCNK2 | Potassium channel, subfamily K, member 2 |
| KCNQ3 | Potassium voltage-gated channel, KQT-like subfamily, member 3 |
| MAGI1 | Membrane associated guanylate kinase, WW and PDZ domain containing 1 |
| MAOA | Monoamine oxidase A |
| MARK1 | MAP/microtubule affinity-regulating kinase 1 |
| MDM2 | Mdm2 p53 binding protein homolog (mouse) |
| MGMT | O-6-methylguanine-DNA methyltransferase |
| MLH1 | Mutl homolog 1, colon cancer, nonpolyposis type 2 (E. Coli) |
| MMP12 | Matrix metallopeptidase 12 (macrophage elastase) |
| MMP3 | Matrix metallopeptidase 3 (stromelysin 1, progelatinase) |
| MTHFR | 5,10-methylenetetrahydrofolate reductase (NADPH) |
| MTRR | 5-methyltetrahydrofolate-homocysteine methyltransferase reductase |
| MTUS1 | Mitochondrial tumor suppressor 1 |
| NAT1 | N-acetyltransferase 1 (arylamine N-acetyltransferase) |
| NAT2 | N-acetyltransferase 2 (arylamine N-acetyltransferase) |
| NBN | Nibrin |
| NCS1 | Frequenin homolog (Drosophila) |
| NPPA | Natriuretic peptide precursor A |
| NPSR1 | Neuropeptide S receptor 1 |
| NPY1R | Neuropeptide Y receptor Y1 |
| NPY2R | Neuropeptide Y receptor Y2 |
| NR4A2 | Nuclear receptor subfamily 4, group A, member 2 |
| NRXN3 | Neurexin 3 |
| OSBPL1A | Oxysterol binding protein-like 1A |
| PARD3 | Par-3 partitioning defective 3 homolog (C. Elegans) |
| PDCD5 | Programmed cell death 5 |
| PDE4D | Phosphodiesterase 4D, camp-specific (phosphodiesterase E3 dunce homolog, Drosophila) |
| PLAUR | Plasminogen activator, urokinase receptor |
| PON1 | Paraoxonase 1 |
| PRKDC | Protein kinase, DNA-activated, catalytic polypeptide |
| PSMA4 | Proteasome (prosome, macropain) subunit, alpha type, 4 |
| PTPRN2 | Protein tyrosine phosphatase, receptor type, N polypeptide 2 |
| SH3BP5 | SH3-domain binding protein 5 (BTK-associated) |
| SLC6A3 | Solute carrier family 6 (neurotransmitter transporter, dopamine), member 3 |
| SLCO3A1 | Solute carrier organic anion transporter family, member 3A1 |
| SMYD3 | SET and MYND domain containing 3 |
| THSD4 | Thrombospondin, type I, domain containing 4 |
| TPH2 | Tryptophan hydroxylase 2 |
| TRIO | Triple functional domain (PTPRF interacting) |
| TRPC7 | Transient receptor potential cation channel, subfamily C, member 7 |
| TTC12 | Tetratricopeptide repeat domain 12 |
| UCP2 | Uncoupling protein 2 (mitochondrial, proton carrier) |
| UGT2B10 | UDP glucuronosyltransferase 2 family, polypeptide B10 |
| USH2A | Usher syndrome 2A (autosomal recessive, mild) |
| VPS13A | Vacuolar protein sorting 13 homolog A (S. Cerevisiae) |
| XPC | Xeroderma pigmentosum, complementation group C |
| XRCC1 | X-ray repair complementing defective repair in Chinese hamster cells 1 |
| XRCC3 | X-ray repair complementing defective repair in Chinese hamster cells 3 |
| ZCCHC14 | Zinc finger, CCHC domain containing 14 |
