# Supplementary material for: Pathways and Networks-Based Analysis of Candidate Genes Associated with Nicotine Addiction
Source: PLoS One. 2015 May 12;10(5):e0127438. doi: 10.1371/journal.pone.0127438 (PMC4429103; doi:10.1371/journal.pone.0127438)
Supplement: S2 Table — (DOC) [file pone.0127438.s002.doc]

**S2 Table. Pathways significantly enriched in NAGenes.**

| **Ingenuity Canonical Pathways** | **P-value^a^** | **P_BH_-value^b^** | **NAGenes included** |
| --- | --- | --- | --- |
| cAMP-mediated signaling | 6.31×10^-17^ | 2.00×10^-14^ | ADRA2A, ADRB2, AGTR1, AKAP13, CAMK4, CHRM1, CHRM2, CHRM5, CNR1, CREB1, DRD1, DRD2, DRD3, DRD4, DRD5, GABBR1, GABBR2, GNAS, GRM7, HTR1F, HTR6, NPY1R, OPRM1, PDE4D, RAPGEF3 |
| Calcium Signaling | 1.00×10^-15^ | 2.00×10^-13^ | CAMK4, CHRNA1, CHRNA10, CHRNA2, CHRNA3, CHRNA4, CHRNA5, CHRNA6, CHRNA7, CHRNB1, CHRNB2, CHRNB3, CHRNB4, CHRND, CHRNG, CREB1, GRIK1, GRIN2A, GRIN2B, GRIN3A, ITPR2, TRPC7 |
| G-Protein Coupled Receptor Signaling | 2.51×10^-15^ | 3.16×10^-13^ | ADRA2A, ADRB2, AGTR1, CAMK4, CHRM1, CHRM2, CHRM5, CNR1, CREB1, DRD1, DRD2, DRD3, DRD4, DRD5, GABBR1, GABBR2, GNAS, GRM7, HTR1F, HTR2A, HTR6, NPY1R, OPRM1, PDE4D, RAPGEF3 |
| Dopamine Receptor Signaling | 3.16×10^-14^ | 2.51×10^-12^ | COMT, DRD1, DRD2, DRD3, DRD4, DRD5, GNAS, MAOA, MAOB, NCS1, PPP1R1B, PPP2R2B, SLC18A2, SLC6A3, TH |
| Xenobiotic Metabolism Signaling | 2.00×10^-12^ | 1.41×10^-10^ | ABCB1, AHR, CAMK4, CYP1A1, CYP2B6, FMO1, GSTM1, GSTM3, GSTP1, GSTT1, IL6, MAOA, MAOB, MAP3K4, MGMT, NOS2, NQO1, PPP2R2B, SOD3, SULT1A1, TNF, UGT1A9, UGT2B10 |
| Dopamine-DARPP32 Feedback in  cAMP Signaling | 2.69×10^-10^ | 1.45×10^-8^ | CAMK4, CREB1, DRD1, DRD2, DRD3GRIN3A, DRD4, DRD5, GNAS, GRIN2A, GRIN2B, ITPR2, KCNJ6, PPP1R1B, PPP2R2B, PRKG1 |
| Aryl Hydrocarbon Receptor Signaling | 2.88×10^-10^ | 1.45×10^-8^ | AHR, CCND1, CHEK2, CYP1A1, ESR1, GSTM1, GSTM3, GSTP1, GSTT1, IL6, MDM2, NQO1, TGFB1, TNF, TP53 |
| LPS/IL-1 Mediated Inhibition of  RXR Function | 4.37×10^-10^ | 1.78×10^-8^ | ABCB1, ABCC4, APOE, CD14, CETP, CYP2A6, CYP2B6, FMO1, GSTM1, GSTM3, GSTP1, GSTT1, MAOA, MAOB, MGMT, SOD3, SULT1A1, TNF |
| Gαi Signaling | 4.57×10^-10^ | 1.78×10^-8^ | ADRA2A, AGTR1, CHRM2, CNR1, DRD2, DRD3, DRD4, GABBR1, GABBR2, GNAS, GRM7, HTR1F, NPY1R, OPRM1 |
| Superpathway of Melatonin Degradation | 3.72×10^-9^ | 1.32×10^-7^ | CYP1A1, CYP2A6, CYP2B6, CYP2D6, MAOA, MAOB, MPO, SULT1A1, UGT1A9, UGT2B10 |
| Serotonin Receptor Signaling | 6.17×10^-9^ | 1.95×10^-7^ | HTR2A, HTR6, MAOA, MAOB, SLC18A2, SLC6A4, TPH1, TPH2 |
| eNOS Signaling | 1.17×10^-8^ | 3.39×10^-7^ | CAMK4, CHRNA10, CHRNA3, CHRNA4, CHRNA5, CHRNB1, CHRNB4, ESR1, GNAS, HSPA4, ITPR2, NOS3, PRKG1 |
| Glucocorticoid Receptor Signaling | 3.89×10^-8^ | 1.05×10^-6^ | ADRB2, CCNH, CREB1, ERCC2, ESR1, HSPA4, ICAM1, IFNG, IL13, IL6, IL8, NOS2, NPPA, NR3C1, PTGS2, TGFB1, TNF |
| Glutamate Receptor Signaling | 5.13×10^-8^ | 1.29×10^-6^ | CAMK4, DLG4, GRIK1, GRIK2, GRIN2A, GRIN2B, GRIN3A, GRM7, SLC1A2 |
| Neuropathic Pain Signaling In Dorsal  Horn Neurons | 5.50×10^-7^ | 1.29×10^-5^ | BDNF, CAMK4, CREB1 , GRIN2A, GRIN2B, GRIN3A, GRM7, ITPR2, KCNQ3, NTRK2 |
| AMPK Signaling | 1.10×10^-6^ | 2.40×10^-5^ | ADRA2A, ADRB2, CHRNA10, CHRNA3, CHRNA4, CHRNA5, CHRNB1, CHRNB4, GNAS, NOS3, PPP2R2B |
| Hepatic Cholestasis | 1.58×10^-6^ | 3.09×10^-5^ | ABCB1, CD14, CETP, ESR1, GNAS, IFNG, IL6, IL8, MAP3K4, SLCO3A1, TNF |
| Gαs Signaling | 1.58×10^-6^ | 3.09×10^-5^ | ADRB2, CHRM1, CHRM5, CNR1, CREB1, DRD1, DRD5, GNAS, HTR6, RAPGEF3 |
| GABA Receptor Signaling | 1.95×10^-6^ | 3.47×10^-5^ | DNM1, GABARAP, GABBR1, GABBR2, GABRA2, GABRA4, GABRE |
| PXR/RXR Activation | 2.00×10^-6^ | 3.47×10^-5^ | ABCB1, CYP2A6, CYP2B6, GSTM1, IL6, NR3C1, TNF, UGT1A9 |
| Colorectal Cancer Metastasis Signaling | 2.57×10^-6^ | 4.37×10^-5^ | ARRB1, CCND1, GNAS, IFNG, IL6, MLH1, MMP12, MMP3, NOS2, PTGS2, RHOA, TGFB1, TNF, TP53 |
| Role of Cytokines in Mediating  Communication between Immune Cells | 5.75×10^-6^ | 8.71×10^-5^ | IFNG, IL13, IL15, IL6, IL8, TGFB1, TNF |
| Melatonin Degradation I | 5.75×10^-6^ | 8.71×10^-5^ | CYP1A1, CYP2A6, CYP2B6, CYP2D6, SULT1A1, UGT1A9, UGT2B10 |
| Dopamine Degradation | 1.07×10^-5^ | 1.55×10^-4^ | ALDH2, COMT, MAOA, MAOB, SULT1A1 |
| Nicotine Degradation II | 1.15×10^-5^ | 1.55×10^-4^ | CYP1A1, CYP2A6, CYP2B6, CYP2D6, FMO1, UGT1A9, UGT2B10 |
| Serotonin Degradation | 1.15×10^-5^ | 1.55×10^-4^ | ADH1B, ALDH2, MAOA, MAOB, SULT1A1, UGT1A9, UGT2B10 |
| Corticotropin Releasing Hormone Signaling | 1.35×10^-5^ | 1.78×10^-4^ | BDNF, CAMK4, CNR1, CREB1, GNAS, ITPR2, NOS2, NOS3, PTGS2 |
| CDK5 Signaling | 1.55×10^-5^ | 1.95×10^-4^ | BDNF, DRD1, DRD5, GNAS, LAMA1, NTRK2, PPP1R1B, PPP2R2B |
| DNA Double-Strand Break Repair by  Non-Homologous End Joining | 2.24×10^-5^ | 2.57×10^-4^ | MRE11A, NBN, PRKDC, XRCC1 |
| LXR/RXR Activation | 2.34×10^-5^ | 2.57×10^-4^ | APOE, C4A/C4B, CD14, CETP, IL6, NOS2, PON1, PTGS2, TNF |
| Atherosclerosis Signaling | 2.34×10^-5^ | 2.57×10^-4^ | APOE, ICAM1, IFNG, IL6, IL8, MMP3, PON1, TGFB1, TNF |
| Graft-versus-Host Disease Signaling | 2.34×10^-5^ | 2.57×10^-4^ | HLA-B, HLA-DQA1, HLA-DRB1, IFNG, IL6, TNF |
| T Helper Cell Differentiation | 2.63×10^-5^ | 2.82×10^-4^ | HLA-DQA1, HLA-DRB1, IFNG, IL13, IL6, TGFB1, TNF |
| Protein Kinase A Signaling | 2.82×10^-5^ | 2.95×10^-4^ | ACP1, AKAP13, ANAPC1, CAMK4, CREB1, GNAS, ITPR2, NOS3, PDE4D, PPP1R1B, PTEN, PTGS2, PTPRD, RHOA, TGFB1, TH |
| Superoxide Radicals Degradation | 3.89×10^-5^ | 3.89×10^-4^ | NQO1, SOD2, SOD3 |
| Nicotine Degradation III | 4.79×10^-5^ | 4.68×10^-4^ | CYP1A1, CYP2A6, CYP2B6, CYP2D6, UGT1A9, UGT2B10 |
| Hepatic Fibrosis / Hepatic Stellate  Cell Activation | 6.92×10^-5^ | 6.61×10^-4^ | AGTR1, CD14, ICAM1, IFNG, IL6, IL8, LAMA1, TGFB1, TNF |
| Nucleotide Excision Repair Pathway | 7.41×10^-5^ | 6.92×10^-4^ | CCNH, ERCC2, ERCC6, RAD23B, XPC |
| Noradrenaline and Adrenaline Degradation | 8.51×10^-5^ | 7.76×10^-4^ | ADH1B, ALDH2, COMT, MAOA, MAOB |
| NRF2-mediated Oxidative Stress Response | 1.00×10^-4^ | 8.91×10^-4^ | ABCC4, EPHX1, FMO1, GSTM1, GSTM3, GSTP1, GSTT1, NQO1, SOD2, SOD3 |
| ATM Signaling | 1.10×10^-4^ | 9.12×10^-4^ | CHEK2, CREB1, MDM2, MRE11A, NBN, TP53 |
| Altered T Cell and B Cell Signaling in  Rheumatoid Arthritis | 1.10×10^-4^ | 9.12×10^-4^ | HLA-DQA1, HLA-DRB1, IFNG, IL15, IL6, TGFB1, TNF |
| Hereditary Breast Cancer Signaling | 1.15×10^-4^ | 9.33×10^-4^ | CCND1, CHEK2, MLH1, MRE11A, NBN, PTEN, TP53, XPC |
| Bladder Cancer Signaling | 1.35×10^-4^ | 1.05×10^-3^ | CCND1, FGF12, IL8, MDM2, MMP12, MMP3, TP53 |
| IL-8 Signaling | 1.38×10^-4^ | 1.05×10^-3^ | ARRB2, CCND1, GNAS, ICAM1, IL8, ITGB3, MPO, PTGS2, RHOA, TEK |
| ILK Signaling | 1.38×10^-4^ | 1.05×10^-3^ | ACTN1, CCND1, CREB1, ITGB3, NOS2, PPP2R2B, PTEN, PTGS2, RHOA, TNF |
| Synaptic Long Term Potentiation | 1.45×10^-4^ | 1.07×10^-3^ | CAMK4, CREB1, GRIN2A, GRIN2B, GRIN3A, GRM7, ITPR2, RAPGEF3 |
| Communication between Innate and  Adaptive Immune Cells | 1.55×10^-4^ | 1.15×10^-3^ | HLA-B, HLA-DRB1, IFNG, IL15, IL6, IL8, TNF |
| Hypoxia Signaling in the Cardiovascular System | 1.86×10^-4^ | 1.32×10^-3^ | CREB1, MDM2, NOS3, NQO1, PTEN, TP53 |
| Leukocyte Extravasation Signaling | 2.19×10^-4^ | 1.55×10^-3^ | CTNNA2, ICAM1, MMP3, RHOA, CTNNA3, RAPGEF3, MAP3K4, MMP12, ACTN1, ITGB3 |
| Role of Hypercytokinemia/hyperchemokinemia  in the Pathogenesis of Influenza | 2.24×10^-4^ | 1.55×10^-3^ | IFNG, IL15, IL6, IL8, TNF |
| Cellular Effects of Sildenafil (Viagra) | 2.40×10^-4^ | 1.62×10^-3^ | CAMK4, GNAS, ITPR2, KCNQ3, NOS3, NPPA, PDE4D, PRKG1 |
| Amyotrophic Lateral Sclerosis Signaling | 2.63×10^-4^ | 1.74×10^-3^ | GRIK1, GRIK2, GRIN2A, GRIN2B, GRIN3A, SLC1A2, TP53 |
| Bupropion Degradation | 3.02×10^-4^ | 1.95×10^-3^ | CYP1A1, CYP2A6, CYP2B6, CYP2D6 |
| nNOS Signaling in Neurons | 3.09×10^-4^ | 2.00×10^-3^ | CAMK4, DLG4, GRIN2A, GRIN2B, GRIN3A |
| HIF1α Signaling | 3.31×10^-4^ | 2.09×10^-3^ | EGLN2, MDM2, MMP12, MMP3, NOS2, NOS3, TP53 |
| Acetone Degradation I (to Methylglyoxal) | 3.47×10^-4^ | 2.14×10^-3^ | CYP1A1, CYP2A6, CYP2B6, CYP2D6 |
| CREB Signaling in Neurons | 3.98×10^-4^ | 2.40×10^-3^ | CAMK4, CREB1, GNAS, GRIK1, GRIK2, GRIN2A, GRIN2B, GRM7, ITPR2 |
| Glutathione-mediated Detoxification | 3.98×10^-4^ | 2.40×10^-3^ | GSTM1, GSTM3, GSTP1, GSTT1 |
| Production of Nitric Oxide and Reactive  Oxygen Species in Macrophages | 4.68×10^-4^ | 2.75×10^-3^ | APOE, IFNG, MAP3K4, MPO, NOS2, PON1, PPP2R2B, RHOA, TNF |
| Endometrial Cancer Signaling | 5.01×10^-4^ | 2.88×10^-3^ | CCND1, CTNNA2, MLH1, PTEN, TP53 |
| Role of Tissue Factor in Cancer | 5.25×10^-4^ | 2.95×10^-3^ | ARRB1, ARRB2, IL8, ITGB3, PLAUR, PTEN, TP53 |
| Role of Macrophages, Fibroblasts and  Endothelial Cells in Rheumatoid Arthritis | 5.25×10^-4^ | 2.95×10^-3^ | CAMK4, CCND1, CREB1, ICAM1, IL15, IL6, IL8, MMP3, NOS2, RHOA, TGFB1, TNF |
| Gαq Signaling | 6.03×10^-4^ | 3.31×10^-3^ | AGTR1, CAMK4, CHRM1, CHRM5, GNAS, HTR2A, ITPR2, RHOA |
| Prostate Cancer Signaling | 6.46×10^-4^ | 3.47×10^-3^ | CCND1, CREB1, GSTP1, MDM2, PTEN, TP53 |
| Role of CHK Proteins in Cell Cycle  Checkpoint Control | 6.46×10^-4^ | 3.47×10^-3^ | CHEK2, MRE11A, NBN, PPP2R2B, TP53 |
| Phenylalanine Degradation IV (Mammalian,  via Side Chain) | 6.61×10^-4^ | 3.47×10^-3^ | ALDH2, MAOA, MAOB |
| Circadian Rhythm Signaling | 8.51×10^-4^ | 4.47×10^-3^ | CREB1, GRIN2A, GRIN2B, GRIN3A |
| TREM1 Signaling | 8.91×10^-4^ | 4.57×10^-3^ | ICAM1, IL6, IL8, MPO, TNF |
| Airway Inflammation in Asthma | 9.33×10^-4^ | 4.68×10^-3^ | IL13, TNF |
| Melatonin Degradation II | 9.33×10^-4^ | 4.68×10^-3^ | MAOB, MAOA |
| Role of BRCA1 in DNA Damage Response | 9.55×10^-4^ | 4.68×10^-3^ | CHEK2, MLH1, MRE11A, NBN, TP53 |
| PI3K/AKT Signaling | 9.77×10^-4^ | 4.68×10^-3^ | CCND1, MDM2, NOS3, PPP2R2B, PTEN, PTGS2, TP53 |
| Crosstalk between Dendritic Cells and Natural Killer Cells | 9.77×10^-4^ | 4.68×10^-3^ | HLA-B, HLA-DRB1, IFNG, IL15, IL6, TNF |
| p53 Signaling | 1.17×10^-3^ | 5.50×10^-3^ | CCND1, CHEK2, MDM2, PRKDC, PTEN, TP53 |
| Antigen Presentation Pathway | 1.17×10^-3^ | 5.50×10^-3^ | HLA-B, HLA-DQA1, HLA-DRB1, IFNG |
| Putrescine Degradation III | 1.20×10^-3^ | 5.50×10^-3^ | ALDH2, MAOA, MAOB |
| Estrogen Biosynthesis | 1.32×10^-3^ | 5.89×10^-3^ | CYP1A1, CYP2A6, CYP2B6, CYP2D6 |
| Differential Regulation of Cytokine Production in Macrophages and T Helper Cells by  IL-17A and IL-17F | 1.41×10^-3^ | 6.31×10^-3^ | IL13, IL6, TNF |
| Tryptophan Degradation X (Mammalian,  via Tryptamine) | 1.41×10^-3^ | 6.31×10^-3^ | ALDH2, MAOA, MAOB |
| Catecholamine Biosynthesis | 1.55×10^-3^ | 6.76×10^-3^ | DBH, TH |
| Citrulline-Nitric Oxide Cycle | 1.55×10^-3^ | 6.76×10^-3^ | NOS2, NOS3 |
| Thyroid Cancer Signaling | 1.58×10^-3^ | 6.76×10^-3^ | BDNF, CCND1, NTRK2, TP53 |
| MIF Regulation of Innate Immunity | 1.74×10^-3^ | 7.41×10^-3^ | CD14, NOS2, PTGS2, TP53 |
| Sertoli Cell-Sertoli Cell Junction Signaling | 1.91×10^-3^ | 7.94×10^-3^ | ACTN1, CTNNA2, MAP3K4, NOS2, NOS3, PRKG1, PTEN, TNF |
| Melanoma Signaling | 1.91×10^-3^ | 7.94×10^-3^ | CCND1, MDM2, PTEN, TP53 |
| GADD45 Signaling | 1.95×10^-3^ | 7.94×10^-3^ | CCND1, MAP3K4, TP53 |
| Dendritic Cell Maturation | 1.95×10^-3^ | 7.94×10^-3^ | CREB1, HLA-B, HLA-DQA1, HLA-DRB1, ICAM1, IL15, IL6, TNF |
| Synaptic Long Term Depression | 2.04×10^-3^ | 8.13×10^-3^ | GNAS, GRM7, ITPR2, NOS2, NOS3, PPP2R2B, PRKG1 |
| IL-17 Signaling | 2.19×10^-3^ | 8.51×10^-3^ | IL6, IL8, MMP3, NOS2, PTGS2 |
| Cardiac Hypertrophy Signaling | 2.24×10^-3^ | 8.71×10^-3^ | ADRA2A, ADRB2, CAMK4, CREB1, GNAS, IL6, MAP3K4, RHOA, TGFB1 |
| iNOS Signaling | 2.29×10^-3^ | 8.71×10^-3^ | CAMK4, CD14, IFNG, NOS2 |
| Cell Cycle: G2/M DNA Damage Checkpoint  Regulation | 2.29×10^-3^ | 8.71×10^-3^ | CHEK2, MDM2, PRKDC, TP53 |
| Serotonin and Melatonin Biosynthesis | 2.29×10^-3^ | 8.71×10^-3^ | TPH1, TPH2 |
| Clathrin-mediated Endocytosis Signaling | 2.51×10^-3^ | 9.33×10^-3^ | APOE, ARRB1, ARRB2, DNM1, FGF12, ITGB3, MDM2, PON1 |
| Type I Diabetes Mellitus Signaling | 2.51×10^-3^ | 9.33×10^-3^ | HLA-B, HLA-DQA1, HLA-DRB1, IFNG, NOS2, TNF |
| HER-2 Signaling in Breast Cancer | 2.75×10^-3^ | 1.00×10^-2^ | CCND1, ITGB3, MDM2, PARD3, TP53 |

1. P-value were calculated by Fisher’s exact test.
2. P_BH_-value were adjusted by Benjamini & Hochberg (BH) method
3. Genes from NAGenes included in the pathway
